# Emotion Recognition Using EEG Signals and Audiovisual Features with Contrastive Learning

**DOI:** 10.3390/bioengineering11100997

**Published:** 2024-10-03

**Authors:** Ju-Hwan Lee, Jin-Young Kim, Hyoung-Gook Kim

**Affiliations:** 1 Department of Intelligent Electronics and Computer Engineering, Chonnam National University, 77 Yongbong-ro, Buk-gu, Gwangju 61186, Republic of Korea; juhwanlee@jnu.ac.kr (J.-H.L.); beyondi@jnu.ac.kr (J.-Y.K.); 2 Department of Electronic Convergence Engineering, Kwangwoon University, 20 Gwangun-ro, Nowon-gu, Seoul 01897, Republic of Korea

**Keywords:** emotion recognition, multimodal learning, contrastive learning, cross-attention mechanism

## Abstract

Multimodal emotion recognition has emerged as a promising approach to capture the complex nature of human emotions by integrating information from various sources such as physiological signals, visual behavioral cues, and audio-visual content. However, current methods often struggle with effectively processing redundant or conflicting information across modalities and may overlook implicit inter-modal correlations. To address these challenges, this paper presents a novel multimodal emotion recognition framework which integrates audio-visual features with viewers’ EEG data to enhance emotion classification accuracy. The proposed approach employs modality-specific encoders to extract spatiotemporal features, which are then aligned through contrastive learning to capture inter-modal relationships. Additionally, cross-modal attention mechanisms are incorporated for effective feature fusion across modalities. The framework, comprising pre-training, fine-tuning, and testing phases, is evaluated on multiple datasets of emotional responses. The experimental results demonstrate that the proposed multimodal approach, which combines audio-visual features with EEG data, is highly effective in recognizing emotions, highlighting its potential for advancing emotion recognition systems.

## 1. Introduction

Emotions are multifaceted psychological phenomena which result from the interaction of both internal cognitive states and external environmental stimuli. They encompass a wide range of physiological, behavioral, and subjective experiences which reflect an individual’s response to a stimulus [1]. These stimuli can include sensory inputs from multimedia content, such as videos and images, which play a crucial role in evoking emotional responses. Platforms like YouTube, Netflix, and TikTok provide users with dynamic audiovisual content which not only conveys information but also serves as a significant medium for emotional engagement. These interactions highlight the complex nature of emotions, which are influenced by both personal dispositions and external influences. As a result, emotion recognition technology has gained importance in various applications, such as personalized content recommendation [2], therapeutic interventions in medical systems [3], and emotion-driven marketing strategies [4], helping to better understand and respond to user emotions in digital environments.

Conventional emotion recognition typically analyzes users’ responses to stimuli, primarily utilizing physiological signal responses (e.g., EEG or ECG) [5,6] and visual behavioral responses (e.g., facial expressions and voice) [7,8]. Physiological signals offer the advantage of objectively capturing unconscious emotional reactions, while visual behavioral responses enable noninvasive and real-time analysis based on users’ external reactions. An alternative approach to emotion recognition involves analyzing the stimulus itself and recognizing emotions by examining the characteristics of multimedia content [9,10]. The audio-visual signals in multimedia content provide powerful emotional stimuli. For instance, dark lighting and tense background music in horror movies induce fear and anxiety, while bright color schemes and upbeat music in comedy programs evoke joy and laughter. These intrinsic emotions can typically be inferred from visual information (color, brightness, and movement) and audio information (voice energy, frequency patterns, and volume), which is a widely recognized direct emotion recognition method in video emotion recognition [11,12,13].

Recently, multimodal emotion recognition [14,15], which allows for richer information utilization than stimulus- and response-based single-modality emotion recognition, has gained attention. This approach enables a more sophisticated understanding of emotional states by simultaneously analyzing multiple signals such as physiological responses, visual behavioral responses, and audio-visual signals. It has also been discovered that a more robust emotion recognition model can be acquired through the collaboration of different modalities [16,17]. However, multimodal approaches primarily focus on fusion at the feature level [18,19,20,21,22,23] and decision level [24,25,26,27], which can present challenges in processing redundant and conflicting information between modalities [28]. Additionally, concerns have been raised about potentially overlooking implicit correlations between modalities during the formation of high-dimensional feature vectors [29].

To address these issues, we propose an emotion recognition method which leverages contrastive learning [30] and cross-modal attention. Contrastive learning is a technique which clearly learns the relationships between high-dimensional data by placing similar data closer together and dissimilar data farther apart. Cross-modal attention enhances inter-modality interactions by selectively focusing on important information from different modalities. We propose a multimodal emotion recognition method which utilizes both the emotions inherent in video and audio stimuli and the physiological signals directly experienced by humans in response to these stimuli. For this, we employ audio-visual signals and EEG as physiological signals. EEG has proven to be a powerful tool for capturing changes in emotional states, recently demonstrating significant improvements in emotion recognition performance when combined with deep learning [31]. The choice of physiological signals over nonverbal cues is based on emotion theories [32,33]. Physiological signals, being unconscious bodily changes controlled by the autonomic nervous system, can potentially represent emotions more reliably than voluntary or involuntary facial behaviors.

The contributions of this paper can be summarized as follows:

1. In this paper, a multimodal emotion recognition framework is proposed based on audio-visual signals and EEG signals to consider both response and stimulus signals.
2. We integrate modality-specific networks and temporal convolutional networks (TCNs) into modal encoders to extract spatiotemporal representations of multimodal data while employing contrastive learning to capture intra-modal, inter-modal, and inter-class relationships in a shared embedding space.
3. We utilize cross-modal attention mechanisms to enhance the interactions between the extracted representations and to focus on the most salient information from each modality.
4. We demonstrate the superior performance of our proposed method in emotion recognition through benchmark datasets and our own collected dataset.

The remainder of this paper is organized as follows. Section 2 provides a review of the related work, the proposed method is explained in Section 3, experimental results validating the effectiveness of our emotion recognition approach are presented in Section 4, Section 5 discusses the limitations of this work, and finally, Section 6 concludes this paper and suggests directions for future research.

## 2. Related Work

In this section, we present a concise review of the relevant literature, focusing on recent advancements in the field of emotion recognition. Our discussion is structured around three key areas which form the foundation of our proposed framework: multimodal emotion recognition, contrastive learning, and cross-modal attention.

### 2.1. Multimodal Emotion Recognition

Emotion recognition has evolved significantly from its early focus on unimodal approaches. Initially, researchers explored individual modalities such as facial expression analysis [34], speech signal processing [35], and physiological signal analysis [36]. These unimodal methods provided valuable insights into specific aspects of emotion expression. For instance, EEG-based emotion recognition using hybrid CNN and LSTM models demonstrated promising results in capturing brain activity patterns associated with emotions [37].

However, emotion recognition systems that rely on a single modality often face challenges in real-world environments due to factors such as noise interference and signal degradation. To overcome these limitations, researchers have introduced multimodal emotion recognition techniques which integrate two or more modalities [38,39,40].

Among various multimodal approaches, the fusion of visual and audio data is particularly prevalent. This combination benefits from relatively straightforward data collection and provides complementary information. However, it is important to note that within the visual modality, dynamic stimuli (such as videos) often provide richer emotional information compared with static stimuli (like images). Dynamic visual content captures the temporal evolution of emotions, allowing for a more comprehensive representation of emotional states [41].

Furthermore, the impact of audio, video, and combined audio-video stimuli on emotional responses varies significantly. Audio stimuli can evoke emotions through tone, rhythm, pitch, and acoustic features, while video stimuli provide visual cues such as facial expressions, body language, color schemes, lighting, and movement patterns. When combined, these audio-visual signals in multimedia content serve as powerful emotional stimuli, creating a more immersive experience capable of inducing a wide range of stronger and more complex emotional responses in viewers than either modality alone.

Despite these advantages, multimodal approaches using only visual and auditory signals predominantly capture external emotional expressions, potentially overlooking essential aspects of a person’s internal emotional state. To address this limitation, some researchers have developed frameworks which merge visual or audio data with physiological signals [42,43]. This approach offers the capability to detect subtle or concealed emotions by leveraging both external and internal cues simultaneously.

Further expanding on this concept, some researchers have explored tri-modal systems which integrate visual, audio, and physiological signals simultaneously [44,45]. These approaches assess a more holistic range of emotional indicators, potentially enhancing the sensitivity and robustness of emotion recognition.

Understanding the distinctions between different modalities and the strengths of various modal combinations is crucial for developing more nuanced and effective emotion recognition systems which can adapt to different types of input and scenarios.

### 2.2. Contrastive Learning

Contrastive learning is a self-supervised learning paradigm where a model is trained to learn meaningful representations of input data by contrasting similar and dissimilar examples [46]. While initially developed for unimodal data, particularly in computer vision, its usage has recently expanded to multimodal learning. Contrastive learning has gained prominence in multimodal contexts for several key reasons. First, it is effective in aligning cross-modal data. It enables the mapping of data from different modalities into a shared embedding space. Second, it is highly data-efficient as it can derive valuable representations from unlabeled data, reducing the need for large labeled multimodal datasets and lowering associated costs and time investments. Third, it addresses challenges such as noise and domain bias [47].

Contrastive learning has also been explored in the context of emotion recognition within multimodal frameworks, though this remains an emerging area of study. Dissanayake et al. [48] introduced a method using wearable sensor data to learn representations for individual vital sign modalities via a self-supervised learning mechanism, which are subsequently fused for emotion recognition tasks. Jiang et al. [49] proposed a contrastive learning framework for emotion recognition based on EEG and eye movement data. Similarly, Tang et al. [50] employed a multimodal approach centered on physiological signals, designing emotion encoders which work across domains and modalities, and they introduced a hierarchical network to integrate these modalities in a structured manner. This multimodal contrastive learning approach leverages information from diverse modalities, such as visual, auditory, and EEG data, to enable more precise and robust classification of emotional states. By utilizing the unique emotional features inherent in each modality, it captures richer emotional expressions while compensating for the shortcomings of any single modality, making it more suitable for real-world applications.

However, to the best of our knowledge, no previous studies have been carried out using contrastive learning for emotion classification which combine audiovisual data (especially video) with EEG. We suppose this gap in the research is primarily due to the complexity of the stimuli, particularly when incorporating video content. This complexity presents significant challenges in applying contrastive learning techniques to such diverse and rich modalities simultaneously.

### 2.3. Cross-Modal Attention

Cross-modal attention is a crucial technique in multimodal learning that enables the effective modeling of relationships between different modalities. This mechanism learns how the features from one modality influence those in another, thereby enhancing the interaction and synergy across modalities. For instance, the authors of [51] employed a cross-modal transformer module to capture long-range dependencies between emotional cues and other modal elements in conversational contexts, emphasizing the interaction between text and audio modalities to adaptively promote convergence between them. Similarly, the authors of [52] applied a cross-modal attention mechanism within an audio-visual fusion model for emotion recognition, aiming to learn the correlations between fused feature representations and the representations of individual modalities.

In another approach, the authors of [53] introduced a multimodal method for music emotion recognition, utilizing a cross-modal attention mechanism to integrate information from various modalities into a hierarchical structure. This method efficiently combines the strengths of each modality. Furthermore, the authors of [40] proposed an end-to-end multimodal emotion recognition framework which leverages cross-modal attention to fuse audio and visual data. This approach capitalizes on the complementary properties of different modalities while maintaining modality-specific characteristics, resulting in more discriminative embeddings, more compact within-class representations, and greater separation between classes. Additionally, the authors of [54] adopted a cross-modal attention mechanism for multidimensional emotion recognition, effectively modeling the relationships between audio, visual, and textual modalities. By simultaneously capturing both intra-modality and inter-modality interactions, this approach produces a more refined and sophisticated feature representation.

## 3. Proposed Method

**Figure 1:**
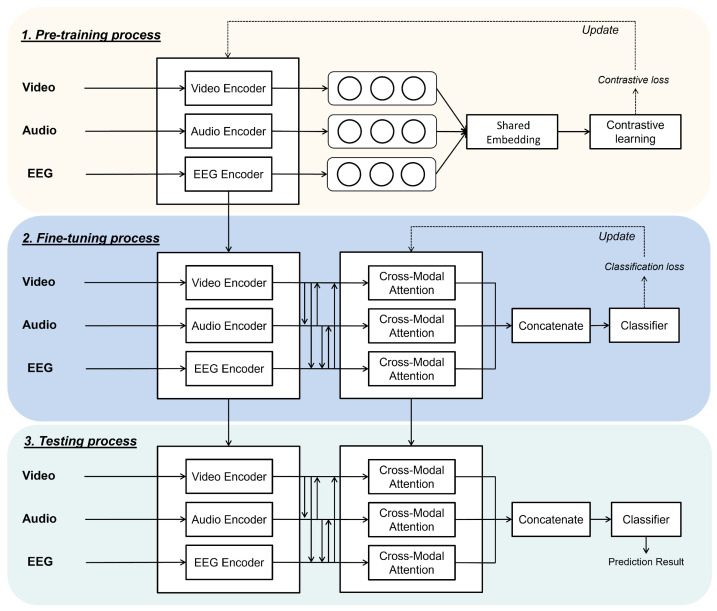
Diagram of a proposed multimodal framework which integrates video, audio, and EEG data for emotion recognition tasks. The framework consists of three main phases: pre-training, fine-tuning, and testing. In the pre-training phase, a modality encoder extracts features and fuses them into a combined embedding using contrastive learning. In the fine-tuning phase, cross-modal attention is utilized to capture interactions between modalities and trained for emotion recogntion. Finally, the test phase is used to obtain predictive results.

In this paper, we present a framework to enhance emotion recognition by leveraging multimodal input data, including visual, audio, and EEG signals. Our approach combines multimodal contrastive learning with cross-modal attention mechanisms to fully exploit both inter-modal and intra-modal relationships, as well as their complementary characteristics. As depicted in Figure 1, the proposed framework consists of three interconnected phases: pre-training, fine-tuning, and testing.

In the pre-training phase, modality-specific encoders are used to extract spatiotemporal features from the visual, audio, and EEG inputs. These features are then optimized in a supervised contrastive learning framework [55] to align the shared embedding spaces of the three modalities, allowing the model to learn more discriminative features for emotion recognition.

The fine-tuning phase incorporates the pre-trained encoders, cross-modal attention modules, and a task-specific classifier. Cross-modal attention is employed to capture and fuse salient feature information across modalities. The classifier is subsequently trained to predict emotion labels based on these multimodal fused representations.

During the testing phase, the fine-tuned encoders and cross-modal attention modules process the input data, and the classifier utilizes the fused multimodal representations to accurately predict emotional states.

### 3.1. Multimodal Encoder

**Figure 2:**
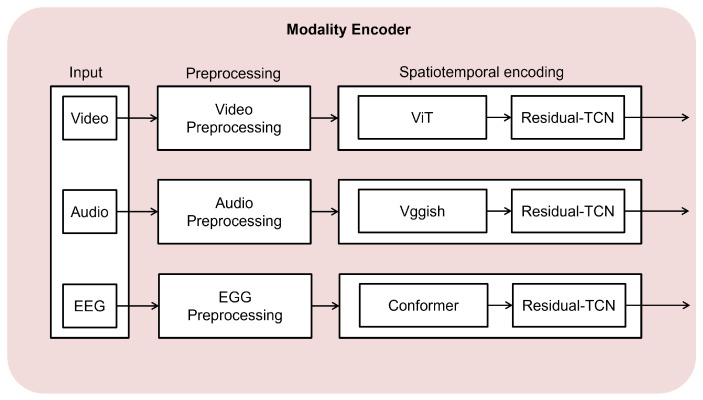
Illustration of modality-specific encoders for extracting spatiotemporal features from video, audio, and EEG data. Each modality is preprocessed before being input to the corresponding encoder: ViT for video, Vggish for audio, and Conformer for EEG. These encoded features are then processed using a Residual-TCN.

This section describes the construction of a multimodal dataset and the encoder architecture used to extract spatiotemporal representations from visual, audio, and EEG data. The overall process involves two main steps: preprocessing and spatiotemporal encoding, as illustrated in Figure 2.

First, we recorded EEG signals while a subject watched videos from a database. The resulting dataset $X={\left\{{\left(x_{v},x_{e},x_{a},y\right)}_{i}\right\}}_{i=1}^{N}$ consisted of *N* samples, where $x_{v}$, $x_{e}$, and $x_{a}$ represent visual, EEG, and audio data, respectively, and *y* is the corresponding label.

In the preprocessing stage, we segmented each video into 4 s blocks with 50% overlap. A demultiplexer separated the audio and visual signals, and we aligned the EEG segments with these blocks to ensure all modalities were synchronized and ready for feature extraction. To address the synchronization of these heterogeneous data types, we specifically applied a time-lagged synchronization approach [56]. This process ensured all modalities were temporally aligned and ready for feature extraction, minimizing any potential impact on the model’s performance due to misalignment.

EEG signals underwent bandpass filtering to extract five distinct frequency bands: delta (0.4–4 Hz), theta (4–8 Hz), alpha (8–13 Hz), beta (13–30 Hz), and gamma (30–45 Hz). For each frequency band, the power spectral density (PSD) was calculated.

Audio signals were processed by applying a 50 ms Hamming window with a 10 ms hop length. This was followed by a short-time Fourier transform (STFT) on each windowed segment to extract the frequency components. The frequency components were then mapped to the Mel scale using 20 Mel filter banks to better align with human auditory perception. Finally, the Mel spectrogram was converted into a log-Mel spectrogram (LMS) by applying a logarithmic function.

For spatiotemporal encoding, we employed modality-specific encoder modules, as shown in Figure 2. Each encoder consisted of two main components: spatial encoding and temporal encoding. The spatial encoding used modality-specific models to capture spatial features, while the temporal encoding utilized a common residual temporal convolutional network (Residual-TCN) across all modalities.

#### 3.1.1. Spatial Encoder

The spatial encoding process aims to extract meaningful spatial features from each modality, considering their unique characteristics. The input to the spatial encoding stage is the preprocessed data for each modality: video frames $x_{v}$, audio Mel spectrograms $x_{a}$, and the EEG power spectral density $x_{e}$.

For the visual data, we used a pre-trained vision transformer (ViT) [57], denoted as $f_{v}$, to extract spatial features from each video frame. The output of the visual spatial encoding, $s_{v}$, had a shape of $R^{T_{v}\times D_{v}}$, where $T_{v}$ is the number of video frames and $D_{v}$ is the feature dimension of the visual spatial encoding.

Similarly, for the audio data, we applied a pre-trained Vggish [58] convolutional neural network, denoted as $f_{a}$, to extract features from the Mel spectrogram. The output of the audio spatial encoding, $s_{a}$, had a shape of $R^{T_{a}\times D_{a}}$, where $T_{a}$ is the number of audio frames and $D_{a}$ is the feature dimension of the audio spatial encoding.

For the EEG data, we used a modified Conformer [59] neural network, denoted as $f_{e}$, to extract spatial features from the power spectral density, excluding the self-attention mechanism. The output of the EEG spatial encoding $s_{e}$ had a shape of $R^{T_{e}\times D_{e}}$, where $T_{e}$ is the number of EEG segments and $D_{e}$ is the feature dimension of the EEG spatial encoding.

The spatial encoding process maps the input data from each modality to a feature space which captures the essential spatial information. The encoded spatial features $s_{v}$, $s_{a}$, and $s_{e}$ serve as the input to the subsequent temporal encoding stage.

#### 3.1.2. Temporal Encoder

After spatial encoding, we employed a Residual-TCN for temporal encoding across all modalities. The Residual-TCN takes the spatially encoded features $s_{v}$, $s_{a}$, and $s_{e}$ as input, extracting the temporal dependencies within each modality. The Residual-TCN effectively extracts and represents the temporal features from the video [60,61,62], audio [63,64], and EEG [65,66,67], enabling it to capture the unique temporal dynamics of each modality.

The network is composed of multiple blocks which process and refine temporal features. Each block begins with a dilated convolution layer, expanding the receptive field to capture long-range dependencies without increasing the parameter count. The dilated convolution is defined as

(1) $$F\left(t\right)=\sum_{i=0}^{k-1}f\left(i\right)\cdot x\left(t-d\cdot i\right)$$

where F(t) is the output at time *t*, *f* is the filter of a size *k*, *x* is the input, and *d* is the dilation rate. This operation allows the network to efficiently capture long-range dependencies by progressively increasing the dilation rate across layers. The dilation rate *d* determines the spacing between input elements in the convolution, enabling the network to expand its receptive field exponentially with depth while maintaining computational efficiency.

After the dilated convolution, a batch normalization layer stabilizes the learning process, followed by an ELU activation function. A dropout layer (rate of 0.5) prevents overfitting, and a residual connection facilitates smooth gradient flow. We stacked four such blocks, progressively increasing the dilation rate (1, 2, 4, and 8) to model both short- and long-term temporal patterns.

The final outputs of the Residual-TCN for visual, audio, and EEG modalities are represented by $h_{v}\in R^{B\times T_{v}\times D}$, $h_{a}\in R^{B\times T_{a}\times D}$, and $h_{e}\in R^{B\times T_{e}\times D}$, where *B* is the batch size, *T* is the sequence length, and *D* is the feature dimension. These representations capture rich spatiotemporal information, forming a strong foundation for the subsequent contrastive learning phase, which enables the model to learn discriminative features for multimodal emotion recognition.

### 3.2. Contrastive Learning for Multimodal Representation

Contrastive learning has rapidly gained traction as an effective technique for aligning the embedding spaces of multimodal data [68,69,70,71]. This approach is particularly effective in multimodal contexts due to its ability to learn shared representations across different modalities, handle heterogeneous data, exploit cross-modal correspondences, and provide robustness to modality-specific noise. By learning discriminative representations, contrastive learning enhances the ability to distinguish between classes by pulling semantically similar samples closer in the latent space and pushing dissimilar samples apart.

**Figure 3:**
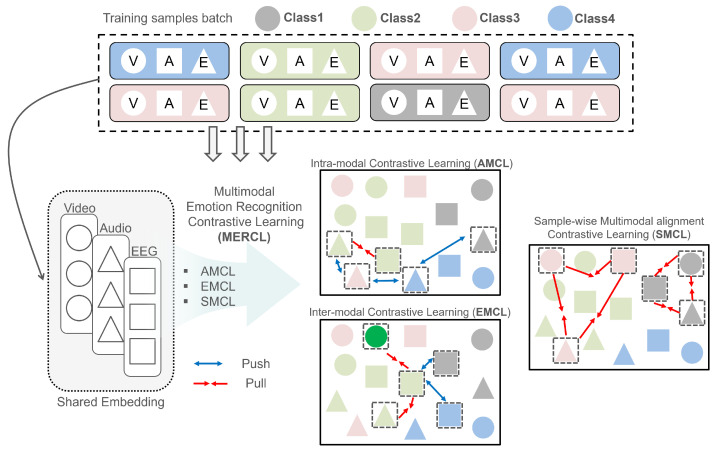
MERCL consists of three components. (1) AMCL learns class-specific relationships within the same modality. (2) EMCL aligns representations across different modalities within the same sample. (3) Finally, SMCL minimizes modality gaps by aligning representations of different modalities within the same sample.

To fully leverage contrastive learning in multimodal emotion recognition, it is essential to account for both intra-modal relationships within each modality and inter-modal interactions across modalities. Intra-modal learning captures the distinct characteristics inherent to each modality, ensuring that the model recognizes subtle patterns specific to visual, audio, or EEG data. Inter-modal learning, on the other hand, focuses on the complementarities between modalities, allowing the model to integrate and enhance shared information across different sources. By addressing both intra- and inter-modal relationships, as illustrated in Figure 3, the model becomes more capable of learning robust and discriminative representations, ultimately improving its ability to accurately classify emotions.

For this phase, we first projected the modality-specific spatiotemporal representations $h_{v}$, $h_{a}$, $h_{e}$ obtained from the encoding module into a shared embedding space by passing them through a projection layer:

(2) $$z_{m}=W_{m}h_{m},\;m\in \left\{v,a,e\right\}$$

where $z_{m}$ represents the projected representation in the shared embedding space for the modality *m*. $W_{m}$ denotes the projection matrix specific to each modality, and $h_{m}$ is the original spatiotemporal representation for that modality.

**(1) Intra-Modal Contrastive Learning (AMCL)**: AMCL focuses on learning class-specific relationships within a single modality by using supervised contrastive learning. For each minibatch, a set $C=\{p_{1}^{m},p_{2}^{m},\cdots ,p_{N}^{m},n_{1}^{m},n_{2}^{m},\cdots ,n_{M}^{m}\}$ containing *N* positive samples and *M* negative samples is generated for a modality *m*. The AMCL loss is defined as

(3) $$L_{AMCL}=-E_{c}\left[\log\frac{\sum_{i=1}^{N}{\left(a^{m}\right)}^{T}p_{i}^{m}}{\sum_{i=1}^{N}{\left(a^{m}\right)}^{T}p_{i}^{m}+\sum_{j=1}^{M}{\left(a^{m}\right)}^{T}n_{j}^{m}}\right],m\in \left\{v,a,e\right\}$$

where $a^{m}$ is the anchor representation and $p_{i}^{m}$ and $n_{j}^{m}$ are the positive and negative samples, respectively, within the same modality *m*. This encourages representations of the same class to cluster closer together and those of different classes to be further apart within each modality.

**(2) Inter-Modal Contrastive Learning (EMCL)**: EMCL focuses on learning relationships across different modalities by using supervised contrastive learning. Unlike AMCL, which defines positive and negative pairs within the same modality, EMCL defines these pairs across different modalities. For each minibatch, a set $C=\{p_{1},p_{2},\cdots ,p_{2N},n_{1},n_{2},\cdots ,n_{2M}\}$ containing 2N positive samples and 2M negative samples is generated for an anchor modality *m*. The 2N positive and 2M negative samples are considered because EMCL explores interactions between the anchor modality and two other modalities, creating more pairings. The EMCL loss is defined as

(4) $$L_{EMCL}=-E_{c}\left[\log\frac{\sum_{i=1}^{2N}{\left(a^{m}\right)}^{T}p_{i}}{\sum_{i=1}^{2N}{\left(a^{m}\right)}^{T}p_{i}+\sum_{j=1}^{2M}{\left(a^{m}\right)}^{T}n_{j}}\right],m\in \left\{v,a,e\right\}$$

where $a^{m}$ is the anchor representation from the modality *m* and $p_{i}$ and $n_{j}$ are the positive and negative samples from other modalities, respectively. This loss encourages representations of the same class to be closer across different modalities while pushing apart representations of different classes.

However, while these two methods effectively capture intra- and inter-modal relationships, they may not fully address significant modality gaps within the same sample. Inspired by [72], we introduce an approach which minimizes this gap by aligning different modalities within the same sample, focusing solely on positive pairs. Negative pairs were excluded to prevent over-separation of modalities, which could lead to the loss of valuable modality-specific information. By aligning modalities with positive pairs, this approach preserves their unique characteristics, ensuring a balanced and informative representation. Therefore, we adopted this approach with a key modification. In our dataset, visual information is consistently available, but audio may be missing in cases like silent scenes in films. To address this, we calculated the energy of the audio signal to determine its presence and exclude samples with low or absent audio energy. This adjustment ensured that our method remains robust and adaptable to the specific characteristics of multimodal datasets.

**(3) Sample-wise Multimodal Alignment Contrastive Learning (SMCL)**: SMCL focuses on minimizing the gap between representations of different modalities within the same sample. It only considers positive pairs, defined as embeddings from different modalities within the same sample. For each minibatch, a set $C=\{p_{1}^{m_{2}},p_{2}^{m_{3}}\}$ is generated, where $m_{1}\neq m_{2}\neq m_{3}$ and $m_{1},m_{2},m_{3}\in \left\{v,a,e\right\}$. SMCL also measures the energy of the audio signal and excludes low-energy samples to ensure robustness when the audio modality is missing or unreliable. The SMCL loss function is defined as

(5) $$L_{SMCL}=E_{c}\left[\frac{1}{2}\sum_{i=1}^{2}{\left|\left|{\left(z^{m}\right)}^{T}p_{i}^{m_{i}}-\alpha \right|\right|}^{2}\right],\;m\in \left\{v,a,e\right\}$$

(6) $$\left[L_{SMCL}=E_{c}\left[\frac{1}{2}\left({\left|\left|{\left(z^{m}\right)}^{T}p_{1}^{m_{1}}-\alpha \right|\right|}^{2}+{\left|\left|{\left(z^{m}\right)}^{T}p_{2}^{m_{2}}-\alpha \right|\right|}^{2}\right)\right]\right]$$

where $z^{m}$ is the anchor representation from the modality *m* and $p_{i}^{m_{i}}$ represents positive pairs from other modalities. A modality margin α accommodates minor differences between modalities while ensuring alignment. Minimizing this loss helps the model align representations across modalities within each sample, effectively reducing the modality gap. Here, *i* is considered to be up to two because SMCL aims to align pairs of modalities within the same sample. This reflects the two different modalities being aligned in each positive pair.

Together, these three loss functions are called multimodal emotion recognition contrastive learning (MERCL) loss and are defined as follows:

(7) $$L_{MERCL}=\lambda_{1}L_{AMCL}+\lambda_{2}L_{EMCL}+\lambda_{3}L_{SMCL}$$

where $\lambda_{1}$, $\lambda_{2}$, and $\lambda_{3}$ are hyperparameters that control the contribution of each loss function.

### 3.3. Cross-Modality Attention and Classifier

Cross-modality attention mechanisms can help two different modalities share the most important parts of each other, exploiting the complementarity between modalities to learn paired feature information and improve emotion recognition performance. In this paper, we employ a pairwise cross-modality attention [73,74,75,76] method to process hidden representations obtained through multimodal contrastive learning and identify deep connections between different modalities. This allows the model to provide a more holistic view of the information for emotion recognition, contributing to better performance.

**Figure 4:**
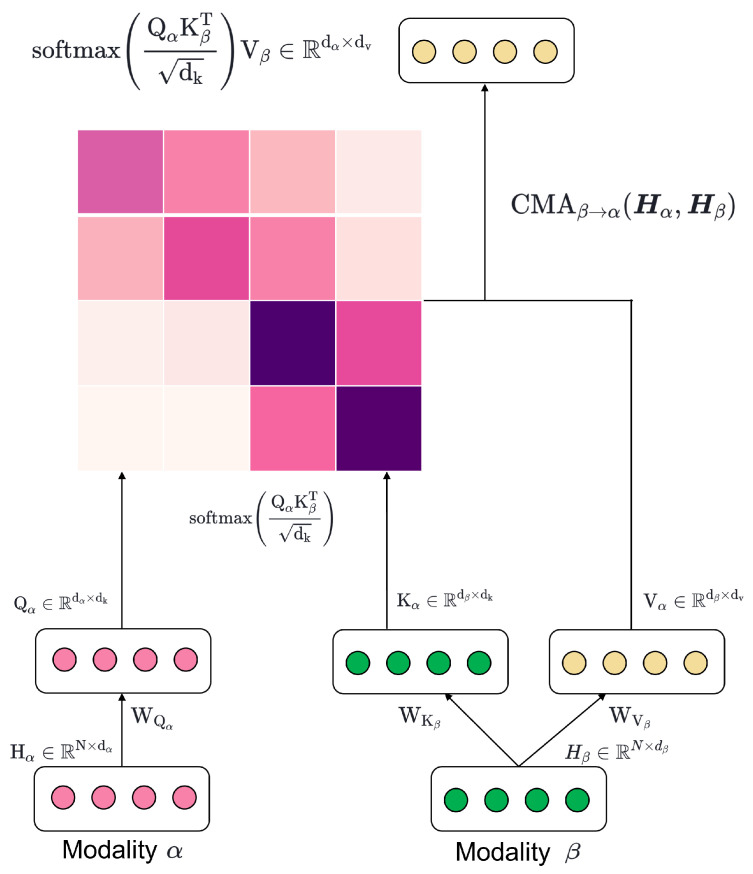
Illustration of the CMA module between modalities α and β.

As depicted in Figure 4, the cross-modal attentions are positioned after fixating the pre-trained encoder, which computes the cross-modality attention (CMA) for the spatiotemporal representations $h_{v}$, $h_{a}$, and $h_{e}$ of each modality output by the encoder. The CMA is calculated using the multi-head attention (MHA) mechanism for each pair of modalities as follows:

- Video-Audio CMA:
  (8) $${\mathrm{CMA}}^{a\to v}=\mathrm{MHA}\left(h_{v},h_{a}\right),\;{\mathrm{CMA}}^{v\to a}=\mathrm{MHA}\left(h_{a},h_{v}\right)$$
- Video-EEG CMA:
  (9) $${\mathrm{CMA}}^{e\to v}=\mathrm{MHA}\left(h_{v},h_{e}\right),\;{\mathrm{CMA}}^{v\to e}=\mathrm{MHA}\left(h_{e},h_{v}\right)$$
- Audio-EEG CMA:
  (10) $${\mathrm{CMA}}^{e\to a}=\mathrm{MHA}\left(h_{a},h_{e}\right),\;{\mathrm{CMA}}^{a\to e}=\mathrm{MHA}\left(h_{e},h_{a}\right)$$

The MHA process allows the model to jointly attend to information from different representation subspaces at different positions. Each head in MHA can focus on different aspects of the input, enabling the model to capture various types of relationships between modalities. The MHA process for each pair of modalities can be described as follows, using ${\mathrm{CMA}}^{a\to v}$ as an example:

(11) $$Q_{i}=h_{v}W_{Q_{i}}^{v},\;K_{i}=h_{a}W_{K_{i}}^{a},\;V_{i}=h_{a}W_{V_{i}}^{a}$$

First, the input vectors $h_{v}$ and $h_{a}$ are linearly transformed into Query, Key, and Value vectors using learnable weight matrices $W_{Q_{i}}^{v}$, $W_{K_{i}}^{a}$, and $W_{V_{i}}^{a}$, respectively. These weight matrices play a crucial role in projecting the input features into different subspaces, allowing the model to capture various aspects of the inter-modal relationships:

(12) $$A_{i}=\frac{Q_{i}K_{i}^{T}}{\sqrt{d_{k}}}V_{i}$$

The attention weights are then calculated by taking the dot product of the Query and Key vectors, scaling the result by the square root of the Key vector’s dimension, and multiplying by the Value vector. This operation allows the model to determine the relevance of each part of the input from one modality to another, effectively capturing the cross-modal interactions:

(13) $${\mathrm{CMA}}^{a\to v}=\mathrm{Concatenation}\left(A_{1},A_{2},...,A_{M}\right)W_{O}$$

The attention outputs from each head are concatenated and linearly transformed using a weight matrix $W_{O}$ to obtain the final multi-head attention result ${\mathrm{CMA}}^{a\to v}$. This combination of multiple attention heads allows the model to capture different types of cross-modal relationships simultaneously, enhancing its ability to understand complex inter-modal interactions. The same process is applied to compute the CMA for the other direction (${\mathrm{CMA}}^{v\to a}$) and for the other pairs of modalities (Video-EEG and Audio-EEG). This bidirectional attention mechanism ensures that the model captures the mutual influence between modalities, rather than just the influence of one modality on another.

The CMA outputs for each pair of modalities ($r_{1},r_{2},...,r_{6}$) are then concatenated into a unified vector $Output_{concatenation}=\left[r_{1},r_{2},...,r_{6}\right]$, which contains the interaction information between all pairs of modalities.

This concatenated output represents a rich, multi-faceted representation of the cross-modal interactions, capturing both the individual modal characteristics and their inter-modal relationships.

Finally, the $Output_{concatenation}$ vector is fed into a multilayer perceptron (MLP) classifier, which consists of a linear layer followed by ReLU activation and a softmax function, to classify the emotion $\hat{y}$. The MLP classifier learns to interpret the complex cross-modal interactions captured by the CMA mechanism, mapping them to emotion categories. This final stage of the model effectively translates the intricate inter-modal relationships into meaningful emotion predictions.

The encoder module, cross-modal attention, and classifier are all optimized through the fine-tuning process using the same dataset used for pre-training. This end-to-end optimization ensures that all components of the model work together coherently to improve emotion recognition performance, leveraging the complementary information from different modalities.

Algorithm 1 presents a concise overview of our proposed multimodal emotion recognition method. This algorithm integrates the pre-training and fine-tuning stages, showcasing the key steps of our approach, including multimodal encoding, projection, contrastive learning, and cross-modal attention.
**Algorithm 1** Multimodal emotion recognition training algorithm.**Require:** Multimodal dataset $D={\left\{{\left(x_{v},x_{a},x_{e},y\right)}_{i}\right\}}_{i=1}^{N}$**Ensure:** Trained model parameters $\theta_{\mathrm{encoder}}$, $\theta_{\mathrm{CMA}}$, $\theta_{\mathrm{classifier}}$  // Stage 1: Pre-training encoders  **for** each pre-training epoch **do**    **for** each mini-batch $(x_{v},x_{a},x_{e})\in D$ **do**        $\left(z_{v},z_{a},z_{e}\right)\leftarrow \mathrm{Encode}\_\mathrm{and}\_\mathrm{Project}\left(x_{v},x_{a},x_{e}\right)$        $L_{\mathrm{MERCL}}\leftarrow \lambda_{1}L_{\mathrm{AMCL}}+\lambda_{2}L_{\mathrm{EMCL}}+\lambda_{3}L_{\mathrm{SMCL}}$        $\theta_{\mathrm{encoder}}\leftarrow \mathrm{Update}\left(\theta_{\mathrm{encoder}},\nabla L_{\mathrm{MERCL}}\right)$  **end for****end for**// Stage 2: Fine-tuning with CMA and classifierFreeze $\theta_{\mathrm{encoder}}$ **for** each fine-tuning epoch **do**    **for** each mini-batch $(x_{v},x_{a},x_{e},y)\in D$ **do**        $\left(h_{v},h_{a},h_{e}\right)\leftarrow \mathrm{Encode}\left(x_{v},x_{a},x_{e}\right)$        // Cross-Modality Attention between modality pairs        ${\mathrm{CMA}}^{a\to v}=\mathrm{MHA}\left(h_{v},h_{a}\right)$, ${\mathrm{CMA}}^{v\to a}=\mathrm{MHA}\left(h_{a},h_{v}\right)$        ${\mathrm{CMA}}^{e\to v}=\mathrm{MHA}\left(h_{v},h_{e}\right)$, ${\mathrm{CMA}}^{v\to e}=\mathrm{MHA}\left(h_{e},h_{v}\right)$        ${\mathrm{CMA}}^{e\to a}=\mathrm{MHA}\left(h_{a},h_{e}\right)$, ${\mathrm{CMA}}^{a\to e}=\mathrm{MHA}\left(h_{e},h_{a}\right)$        Concatenate all CMA outputs:        $Output_{\mathrm{concatenation}}=\left[{\mathrm{CMA}}^{a\to v},{\mathrm{CMA}}^{v\to a},{\mathrm{CMA}}^{e\to v},{\mathrm{CMA}}^{v\to e},{\mathrm{CMA}}^{e\to a},{\mathrm{CMA}}^{a\to e}\right]$        $\hat{y}\leftarrow \mathrm{Classifier}\left(\mathrm{Concatenate}\left(Output_{\mathrm{concatenation}}\right)\right)$        $L_{\mathrm{cls}}\leftarrow \mathrm{Cross}\;\mathrm{entropy}\left(\hat{y},y\right)$        $\theta_{\mathrm{CMA}},\theta_{\mathrm{classifier}}\leftarrow \mathrm{Update}\left(\theta_{\mathrm{CMA}},\theta_{\mathrm{classifier}},\nabla L_{\mathrm{cls}}\right)$    **end for****end for****return** $\theta_{\mathrm{encoder}}$, $\theta_{\mathrm{CMA}}$, $\theta_{\mathrm{classifier}}$

## 4. Experimental Results

In this study, we evaluated the efficacy of our proposed method using four distinct datasets: DEAP [77], SEED [78], DEHBA, and MTIY. These datasets were selected for their comprehensive collection of EEG data elicited by audiovisual emotional stimuli.

### 4.1. Evaluation Datasets

- DEAP: The DEAP dataset contains EEG and peripheral signals collected from 32 participants (16 males and 16 females between the ages of 19 and 37). EEG signals were recorded while each participant watched 40 music video clips. Each participant rated their level of arousal, valence, dominance, and preference on a continuous scale from 1 to 9 using a Self-Assessment Manikin (SAM). Each trial contained 63 s of EEG signals, with the first 3 s serving as the baseline signal. The EEG signals were recorded at a sampling rate of 512 Hz using 32 electrodes. For this study, EEG data from 20 participants (10 males and 10 females) were selected for the experiment.
- SEED: The SEED dataset contains EEG and eye movement signals collected from 15 participants (7 males and 8 females). For this study, data from 10 participants (5 males and 5 females) were selected. Each participant’s EEG signals were collected while watching 15 Chinese movie clips approximately 4 min in length, designed to evoke positive, neutral, and negative emotions. The signals collected from 62 electrodes had a sampling rate of 1 kHz, which was then downsampled to 200 Hz. After watching each film clip, each participant recorded an emotion label for each video as negative (−1), neutral (0), or positive (1).
- DEHBA: The DEHBA dataset is a human EEG dataset collected during emotional audiovisual stimulation. EEG data were measured while subjects watched video clips designed to elicit four emotional states: (1) happy, (2) sad, (3) angry, and (4) relaxed. These states are defined on a plane with axes representing arousal and valence from the circumplex model of affect: “happy” corresponds to high valence and high arousal (HVHA), “angry” corresponds to low valence and high arousal (LVHA) “sad” corresponds to low valence and low arousal (LVLA), and “relaxed” corresponds to high valence and low arousal (HVLA).
  Researchers selected 100 videos (25 for each emotional state) based on their ability to elicit strong emotions without relying on language understanding. These videos were validated by 30 college students, who rated the intensity of their emotions after viewing each clip. EEG data were collected from 30 participants using a 36 channel electrode cap at a sampling rate of 1 kHz, and for this study, data from 12 participants (6 males and 6 females) were selected for analysis.
  The participants reported their emotional responses and rated the intensity of the emotions they experienced after viewing each video. This feedback was used to refine data selection and evaluate the results.
- MTIY: The Movie Trailer In YouTube (MTIY) dataset was constructed from 50 movie trailer videos retrieved from YouTube using the search term “movie trailer”. The videos covered five genres—science fiction, comedy, action, horror, and romance—with 10 videos in each genre, and each video was 60 s long. Subjects were instructed to watch all 50 videos, and an Emotiv headset was used to obtain EEG signals, with EEG features extracted every second. The EEG data were collected using 14 electrodes. The EEG features were collected using 36 electrodes. For this study, data from 16 participants (8 males and 8 females) were used. Each subject rated the level of arousal, valence, dominance, and preference on a continuous scale from 1 to 9 after watching all of the videos.

### 4.2. Experimental Set-Up

To evaluate the emotion recognition performance of the proposed method and compare it with other existing approaches, we employed fourfold cross-validation to ensure robust evaluation. For comparison, we used existing multimodal models with both feature-level fusion and decision-level fusion methods. The following baseline methods were applied in the experiments:

- VE-BiLSTM [79]: This method employs a two-layer bidirectional LSTM network. It performs feature-level fusion by concatenating video and EEG features as input, where the video features are 1024 dimensional and the EEG features are also 1024 dimensional. The first LSTM layer has 1024 hidden units, and the second LSTM layer has 256 hidden units. The final recognition is performed using a softmax layer on top of the concatenated forward and backward hidden states from the second Bi-LSTM layer.
- AVE-RT [45]: This method combines EEG, audio, and visual features for emotion recognition through feature-level fusion. It extracts power spectral density features from the EEG signals across five frequency bands, audio features using eGeMAPS [80], and visual features, including the luminance coefficient and color energy. These multimodal features are concatenated at the feature level and fed into a random tree classifier for emotion recognition.
- AVE-KELM [81]: This method combines video content and EEG signals. It extracts audio-visual features from video clips and EEG features using wavelet packet decomposition (WPD). The video features are selected using double input symmetrical relevance (DISR), while EEG features are selected by a decision tree (DT). The selected features from both modalities are then combined at the decision level using a kernel-based extreme learning machine (ELM) for final emotion recognition.
- AVE-LSTM [82]: This method integrates the audio, video, and EEG modalities for emotion recognition. Each modality has its own feature extractor, and LSTM networks are used for emotion recognition. Specifically, audio features are derived from MFCC, video features are extracted using VGG19, and EEG features are obtained through PCA after bootstrapping. The outputs from each LSTM are individually used for emotion recognition, and the final emotion prediction is achieved through decision fusion of these results. While the original approach also incorporated EMG data, our implementation excluded this modality.

All baseline methods were reimplemented according to the model configurations provided by their respective authors to ensure fair comparison. This approach allowed us to directly compare the performance of our proposed method with existing techniques under consistent experimental conditions.

The performance of each method was evaluated by using the accuracy and F1 score as the evaluation metrics:

(14) $$\mathrm{Accuracy}=\frac{\mathrm{TP}+\mathrm{TN}}{\mathrm{TP}+\mathrm{TN}+\mathrm{FP}+\mathrm{FN}}$$

(15) $$F1=\frac{2\times \mathrm{TP}}{2\times \mathrm{TP}+\mathrm{FP}+\mathrm{FN}}$$

where true positive (TP) is the number of positive samples correctly classified as positive, true negative (TN) is the number of negative samples correctly classified as negative, false positive (FP) is the number of negative samples incorrectly classified as positive, and false negative (FN) is the number of positive samples incorrectly classified as negative.

### 4.3. Experimental Results

This section provides a comprehensive analysis of the experimental results obtained from our proposed multimodal emotion recognition method. Our evaluation focuses on comparing the performance of our approach with that of a model combining EEG and audio-visual features, highlighting the effectiveness of our method. We explored how different modality combinations influence overall performance, revealing the impact of each on the model’s efficacy. Through an ablation study, we demonstrate the significance of the contrastive learning and cross-modal attention mechanisms.

To evaluate our proposed model’s performance, we conducted various experiments by using different emotion classification schemes for each dataset. Table 1 and Table 2 illustrate our classification schemes for two-level and three-level emotions, respectively, as applied to the DEAP dataset. For the DEAP dataset, we redefined the emotion classes based on the original 1–9 rating scale for valence, arousal, and dominance. In addition to the two-level and three-level classifications shown in the tables, we implemented a four-level emotion classification by combining the valence and arousal dimensions. This resulted in four categories: HVHA, LVHA, LVLA, and HVLA. The SEED dataset, with its preexisting labels of negative (−1), neutral (0), and positive (1), was used for three-level valence classification experiments without any relabeling. For both the DEHBA and MTIY datasets, we employed the same four-level emotion classification scheme as that used with the DEAP dataset, categorizing emotions into HVHA, LVHA, LVLA, and HVLA based on the combination of valence and arousal dimensions.

Table 3, Table 4, Table 5 and Table 6 show the results of 2–4 levels of emotion classification using the four datasets. The final classification performance results were obtained by calculating the average of all the cross-validation folds.

As shown in the results, the accuracy of emotion classification gradually decreased as the number of classes increased from two to four in the DEAP, SEED, DEBHA, and MITY datasets. This trend aligns with the expectation that as the number of emotion classes to be distinguished increases, the complexity of the patterns that the model needs to learn also increases.

The multimodal emotion recognition method proposed in this study, utilizing contrastive learning and cross-modal attention, consistently demonstrated superior performance compared with existing approaches. As can be seen in Table 3, Table 4, Table 5 and Table 6, the proposed method achieved the highest accuracy and F1 scores across all datasets and classification levels. It should be noted that the higher performance in four-level classification for the DEBHA and MITY datasets compared with the DEAP and SEED datasets can be attributed to the selection of videos with more distinct emotional content in the DEBHA and MITY datasets. This clarity in emotional stimuli resulted in relatively consistent recognition performance across all methods except for our proposed approach, which showed significant improvement.

Existing feature-level fusion approaches (VE-BiLSTM and AVE-RT) simply concatenate features from multiple modalities, but this has limitations in fully capturing the complex interactions between modalities. This limitation becomes more apparent as the number of emotion classes increases. Another approach, decision-level fusion (e.g., AVE-KELM), processes each modality independently and combines them in the final decision stage. While this method can be computationally efficient, it has the drawback of potentially missing early interactions between modalities. In contrast, our proposed method can more effectively capture implicit correlations between modalities by explicitly learning the relationships between the features of each modality through contrastive learning.

Notably, the proposed method showed relatively less performance degradation as the number of classes increased. For example, when transitioning from two-level classification (Table 3) to four-level classification (Table 5) in the DEAP dataset, our method only showed about a 10% decrease in accuracy. In contrast, other methods showed a larger performance drop of 15–20%. This suggests that our approach is robust even in distinguishing more fine-grained emotional states.

Even compared with recent deep learning-based methods like AVE-LSTM, our method consistently showed better performance. This emphasizes that in multimodal emotion recognition, learning the unique characteristics and correlations between modalities is more important than simply using deep neural networks. Lastly, it is noteworthy that our method showed balanced performance improvement across all three emotional dimensions: valence, arousal, and dominance. This indicates that our approach can effectively capture the multidimensional nature of emotions.

We argue that combining external emotional stimuli (audiovisual data) with internal physiological responses (EEG signals) can provide a more comprehensive understanding of emotional states. To clearly understand this, we examined the effects of various modality combinations and investigated how audiovisual signals and EEG, which are in a stimulus–response relationship, interact to improve recognition accuracy.

Accordingly, in Table 7, the experimental results showed that emotion recognition based solely on audiovisual information has limitations in terms of accuracy and robustness. In contrast, when audio or video data were combined with EEG signals representing physiological responses, recognition performance significantly improved. This emphasizes the complementary role of external stimuli and internal physiological reactions in emotion recognition. Notably, among dual-modality combinations, the pairing of video data and EEG signals yielded the best performance, suggesting that visual cues provide particularly valuable information when combined with physiological data.

The most notable point is that the highest accuracy in emotion recognition was achieved when all three modalities—audio, video, and EEG—were integrated. This result demonstrates the effectiveness of learning representations that incorporate both external emotional stimuli and internal physiological responses. These findings showcase the synergistic effect of combining multiple modalities, particularly the integration of external emotional stimuli (audiovisual data) with internal physiological responses (EEG signals). This approach provides a more comprehensive and accurate method for understanding and recognizing complex emotions.

While the modality combination experiments demonstrate the potential of a multi-modal approach, they do not explain how the specific mechanisms of our proposed method maximize this potential. To explore this, we analyzed the contribution of each component through an ablation study. According to the experimental results in Table 8, performance dropped by 4.04% when contrastive learning was removed and by 2.19% when cross-modal attention was removed. When both elements were removed, the performance decreased the most (5.28%). This suggests that contrastive learning has a greater impact on learning rich information from multiple modalities compared with cross-modal attention.

The importance of contrastive learning appears to stem from its ability to effectively capture complex relationships between different modalities and learn an integrated feature space. In particular, contrastive learning can be interpreted as playing a crucial role in learning subtle correlations between internal physiological responses like EEG signals and external expressions like audio-visual data.

The fact that performance dropped the most when both elements were removed shows that contrastive learning and cross-modal attention create a synergistic effect, maximizing the performance of multimodal emotion recognition. While contrastive learning learns the overall relationships between modalities, cross-modal attention enables more fine-grained information exchange based on this, thereby enhancing the model’s expressiveness. These results demonstrate that our proposed method reaches beyond simply combining information from multiple modalities, effectively modeling complex interactions between each modality.

Finally, one important consideration in multimodal learning is how to handle samples that do not contain meaningful information in each modality. These samples can hinder model learning or lead to learning incorrect patterns. This issue becomes even more critical in complex tasks such as emotion recognition.

Table 9 shows our approach to this problem. When applying the audio energy-based sample selection method, the model’s accuracy and F1 score improved. This proves that selectively using samples rich in information is more effective than simply using all samples. The key to this method is selecting samples likely to contain significant information based on the energy level of the audio signal. High-energy audio samples are generally more likely to contain clearer emotional expressions and are expected to have more distinct correlations with other modalities (EEG and video).

## 5. Discussion

While the proposed multimodal emotion recognition framework demonstrated significant improvements in classification accuracy, several important considerations and limitations warrant further discussion and future research.

The integration of multiple modalities and advanced techniques such as contrastive learning and cross-modal attention resulted in a highly complex model. This complexity, while contributing to the model’s performance, poses challenges in terms of interpretability. Developing methods to visualize and explain the model’s internal representations and decision boundaries could provide valuable insights and increase trust in the system’s outputs. Furthermore, investigating how each modality’s signals (video and audio) specifically influence brain responses as captured by EEG data is crucial. This exploration, along with existing efforts in interpreting multimodal emotion recognition systems [83,84,85], could provide insights into the actual interactions between different modalities and their impact on emotional responses. Such research could bridge the gap between computational models and neurophysiological processes, potentially leading to more biologically plausible and interpretable emotion recognition systems.

The current study utilized a limited number of datasets, which may affect the model’s generalizability to diverse populations and contexts. The complex nature of the model, combined with limited data, raises concerns about potential overfitting. It is important to acknowledge the significant challenges in collecting comprehensive datasets for multimodal emotion recognition. Acquiring audiovisual materials which effectively elicit a wide range of emotions, along with corresponding EEG data, is a complex and resource-intensive process. The subjective nature of emotional responses and the variability across individuals further complicate this task. Future work should explore innovative approaches to data collection and augmentation, including semi-supervised learning techniques [86,87] which can leverage limited labeled data more effectively.

The proposed model’s complexity necessitates substantial computational resources for training and inference, which may limit its applicability in real-time or resource-constrained environments. Future research should explore model compression techniques, such as knowledge distillation [88,89,90], to reduce the model’s size and computational requirements without significantly compromising performance. Additionally, investigating incremental learning methods could facilitate more efficient model updates and adaptations to new data, enhancing the model’s practical applicability in dynamic real-world scenarios.

## 6. Conclusions

This study proposed a novel multimodal approach for emotion recognition, integrating audio-visual data with EEG signals. Our research demonstrated that combining externally observable cues with internal physiological responses significantly improves emotion recognition accuracy. The proposed method, utilizing contrastive learning and cross-modal attention, consistently outperformed existing approaches across various datasets and classification levels.

Key findings include the crucial role of EEG signals in enhancing recognition accuracy, particularly when combined with audio-visual data, and the effectiveness of selective sample usage based on audio energy levels. Our approach showed robustness in distinguishing fine-grained emotional states, maintaining relatively high performance even as the number of emotion classes increased.

Future research should focus on enhancing model interpretability, personalizing emotion recognition. Developing techniques to visualize and explain the model’s decision-making process will be crucial, particularly in understanding how different modalities interact and contribute to emotion classification. Investigating adaptive models which account for individual differences in emotional expression and perception could lead to more personalized and accurate systems. Additionally, exploring knowledge distillation methods to create simpler, more efficient models from our complex multimodal approach could address computational constraints while maintaining high performance. These advancements aim to create more interpretable, personalized, and efficient emotion recognition systems suitable for various real-world applications.

## Figures and Tables

**Table 1:** Emotion classes for two-level emotion classification on the DEAP dataset.

Rating Values (RVs)	Valence	Arousal	Dominance
1 ≤ RVs ≤ 5	Low	Low	Low
6 ≤ RVs ≤ 9	High	High	High

**Table 2:** Emotion classes for three-level emotion classification on the DEAP dataset.

Rating Values (RVs)	Valence	Arousal	Dominance
1 ≤ RVs ≤ 3	Negative	Activated	Controlled
4 ≤ RVs ≤ 6	Neutral	Moderate	Moderate
7 ≤ RVs ≤ 9	Positive	Deactivated	Overpowered

**Table 3:** Performance comparison of different methods for two-level classification on the DEAP dataset.

Methods	Valence		Arousal		Dominance	
	**Accuracy**	**F1**	**Accuracy**	**F1**	**Accuracy**	**F1**
VE-BiLSTM [79]	71.8	71.4	70.1	70.2	71.5	71.3
AVE-KELM [81]	78.3	78.1	76.2	76.6	77.9	78.1
AVE-LSTM [82]	82.6	83.1	80.6	80.3	82.1	81.9
AVE-RT [45]	85.7	85.5	82.4	82.2	85.2	84.8
Proposed Method	93.4	93.2	91.7	92.0	93.5	93.2

**Table 4:** Performance comparison of different methods for three-level classification on the DEAP dataset.

Methods	Valence		Arousal		Dominance	
	**Accuracy**	**F1**	**Accuracy**	**F1**	**Accuracy**	**F1**
VE-BiLSTM [79]	64.6	64.2	64.3	63.9	63.7	63.5
AVE-KELM [81]	73.7	73.5	73.2	74.1	72.4	72.1
AVE-LSTM [82]	78.5	77.8	77.1	76.9	76.8	77.2
AVE-RT [45]	80.1	80.3	79.5	80.2	80.7	80.5
Proposed Method	89.3	89.6	88.6	88.2	89.2	89.5

**Table 5:** Performance comparison of different methods for four-level classification on the DEAP dataset and three-level classification on the SEED dataset.

Methods	DEAP: Four-Level		SEED: Three-Level	
	**Accuracy**	**F1**	**Accuracy**	**F1**
VE-BiLSTM [79]	60.1	59.4	69.3	70.2
AVE-KELM [81]	67.5	68.2	75.6	74.9
AVE-LSTM [82]	69.3	70.2	77.3	78.5
AVE-RT [45]	75.5	78.4	81.5	81.3
Proposed Method	83.2	84.1	90.9	91.2

**Table 6:** Performance comparison of different methods for four-level classification on the DEBHA dataset and MITY dataset.

Methods	DEBHA		MITY	
	**Accuracy**	**F1**	**Accuracy**	**F1**
VE-BiLSTM [79]	80.3	80.4	75.6	74.3
AVE-KELM [81]	83.4	82.7	78.3	79.2
AVE-LSTM [82]	85.3	84.1	80.2	81.1
AVE-RT [45]	87.5	86.6	82.6	83.0
Proposed Method	96.5	96.5	91.6	92.7

**Table 7:** Experiment with modality combinations on the DEBHA dataset.

Modality	Accuracy	F1
Audio + Video	78.4	77.8
Audio + EEG	82.5	81.9
Video + EEG	84.6	84.2
Audio + EEG + Video	96.5	96.5

**Table 8:** Ablation study of the impact of contrastive learning and cross-modal attention on the DEBHA dataset.

Condition	Accuracy	F1
Without Contrastive Learning	92.5	91.3
Without Cross-Modal Attention	94.3	94.0
Without Contrastive Learning and Cross-Modal Attention	91.2	92.1
Proposed Method	96.5	96.5

**Table 9:** Impact of audio energy-based sample selection on the DEBHA dataset.

Method	Accuracy	F1
Proposed method (all samples)	96.5	96.3
Proposed method (audio energy-based selection)	97.4	98.1

## Data Availability

The DEAP dataset can be found at https://www.eecs.qmul.ac.uk/mmv/datasets/deap/ (accessed on 5 July 2023). The SEED dataset is available at https://bcmi.sjtu.edu.cn/home/seed/ (accessed on 8 August 2023). Raw data from the DEHBA and MTIY datasets can be obtained by writing a formal email to Hyoung-Gook Kim.
